# The Effects of Intermittent Diet Breaks during 25% Energy Restriction on Body Composition and Resting Metabolic Rate in Resistance-Trained Females: A Randomized Controlled Trial

**DOI:** 10.5114/jhk/159960

**Published:** 2023-01-20

**Authors:** Madelin R. Siedler, Megan H. Lewis, Eric T. Trexler, Priscila Lamadrid, Brian J. Waddell, Sarah F. Bishop, Gillian SanFilippo, Kaitlin Callahan, David Mathas, Gianna F. Mastrofini, Menno Henselmans, Fredrik T. Vårvik, Bill I. Campbell

**Affiliations:** 1 Exercise Science Program, University of South Florida, Tampa, FL, USA.; 2 Kinesiology and Sport Management, Texas Tech University, Lubbock, TX, USA.; 3 Health and Kinesiology, Texas A&M University, College Station, TX, USA.; 4 Exercise Science, University of South Carolina, Columbia, SC, USA.; 5 The International Scientific Research Foundation for Fitness and Nutrition, Amsterdam, The Netherlands.; 6 Sport Science and Physical Education, Faculty of Health and Sport Sciences, University of Agder, Kristiansand, Norway.

**Keywords:** fat mass, physique enhancement, weight loss, muscle mass, metabolism

## Abstract

The purpose of this study was to examine the effects of intermittent versus continuous energy restriction on body composition, resting metabolic rate, and eating behaviors in resistance-trained females. Thirty-eight resistance-trained females (mean ± standard deviation age: 22.3 ± 4.2 years) were randomized to receive either six weeks of a continuous 25% reduction in energy intake (n = 18), or one week of energy balance after every two weeks of 25% energy restriction (eight weeks total; n = 20). Participants were instructed to ingest 1.8 g protein/kilogram bodyweight per day and completed three weekly supervised resistance training sessions throughout the intervention. There were no differences between groups for changes over time in body composition, resting metabolic rate, or seven of the eight measured eating behavior variables (p > 0.05). However, a significant group-by-time interaction for disinhibition (p < 0.01) from the Three-Factor Eating Questionnaire was observed, with values (± standard error) in the continuous group increasing from 4.91 ± 0.73 to 6.17 ± 0.71, while values in the intermittent group decreased from 6.80 ± 0.68 to 6.05 ± 0.68. Thus, diet breaks do not appear to induce improvements in body composition or metabolic rate in comparison with continuous energy restriction over six weeks of dieting, but may be employed for those who desire a short-term break from an energy-restricted diet without fear of fat regain. While diet breaks may reduce the impact of prolonged energy restriction on measures of disinhibition, they also require a longer time period that may be less appealing for some individuals.

## Introduction

Fat loss diets generally involve prolonged periods of negative energy balance, often resulting in decreased levels of fat-free mass (FFM) and resting metabolic rate (RMR); at the same time, as energy restriction continues, perceived levels of hunger tend to rise while satiety signals are reduced (Casanova et al., 2019; Stubbs et al., 2018). The combination of these factors likely generate strong physiological and psychological drives to restore lost weight, making the maintenance of weight loss difficult.

Often, observed decreases in RMR go beyond the reduction that would be predicted based on the loss of body mass alone (Camps et al., 2013; Johannsen et al., 2012; Rosenbaum et al., 2008). These reductions have even been reported to persist for years after the period of energy restriction has ended, even when body mass is partially restored (Fothergill et al., 2016), although full restoration of the prior body composition generally fully reverses any such negative metabolic adaptations (Zinchenko and Henselmans, 2016). Fat-free mass is the largest contributor to RMR (Stubbs et al., 2018), and reductions in FFM as a result of energy restriction may partially explain the prolonged metabolic effects of weight loss. Indeed, one model suggests that in the context of energy balance, the effect of FFM on resulting energy intake is fully mediated by the more direct effect of FFM on RMR (Hopkins et al., 2016). However, it has also been proposed that in the context of FFM loss due to energy restriction, the mediating role of RMR in energy intake may be supplemented or overridden by more direct effects of FFM and fat mass (Stubbs et al., 2018). Observations of hyperphagia during re-feeding after substantial weight loss also suggest that the relationship between FFM recovery and the hyperphagic response exists independently of the recovery of fat stores: while fat stores are more quickly recovered than FFM, the hyperphagic response continues until FFM recovery, leading to a transient period of “body fat overshoot” (Dulloo et al., 1997; Nindl et al., 1997). Thus, efforts to optimize FFM retention during weight loss, such as through the use of concomitant resistance training (Hunter et al., 2008), may help reduce the drive to regain weight after energy restriction has ended.

Finally, energy restriction results in increased perceptions of hunger and decreased perceptions of satiety. This is likely due, at least in part, to hormonal changes including an increase in the concentrations of orexigenic hormones such as ghrelin along with a decrease in anorexigenic hormones such as leptin, peptide YY, and cholecystokinin (Fothergill et al., 2016; Pardina et al., 2009; Sumithran et al., 2011). Furthermore, changes in subjective outcomes such as hunger, desire to eat, and prospective food consumption during weight loss are concordant with these physiological observations (Doucet et al., 2000; Sumithran et al., 2011). Taken together, the effects of energy restriction on changes in FFM, RMR, and appetite all serve to create an environment that promotes weight regain after restriction has ended.

One proposed method of improving the likelihood of weight loss maintenance is the use of intermittent periods of energy balance (or “diet breaks”) throughout a period of energy restriction. While several investigations have examined the use of this approach in individuals with overweight and obesity (Arguin et al., 2012; Byrne et al., 2018; Keogh et al., 2014; Wing and Jeffery, 2003), potential strategies to mitigate the consequences of energy restriction are also highly relevant to leaner individuals, including weight-class and physique athletes who must reduce energy intake in preparation for competitions and events (Peos et al., 2019; Trexler et al., 2014). Until recently, however, no research had been conducted in these populations. In addition, a large portion of the previous investigations on intermittent energy restriction utilized more severe periods of restriction (e.g., alternate-day fasting) coupled with periods of *ad libitum* feeding. However, individuals have been reported to commonly undereat during these periods, thus continuing some degree of energy restriction; this style of intermittent energy restriction may elicit different physiological and psychological effects than an approach in which energy balance is deliberately restored at intermittent periods (Sainsbury et al., 2018). Therefore, the current study aimed to evaluate the effects of intermittent (INT) diet breaks during six weeks of 25% energy restriction versus continuous (CON) restriction to the same degree on body composition, resting metabolic rate, hunger and eating behaviors in young, resistance-trained females.

## Methods

**Figure 1:**
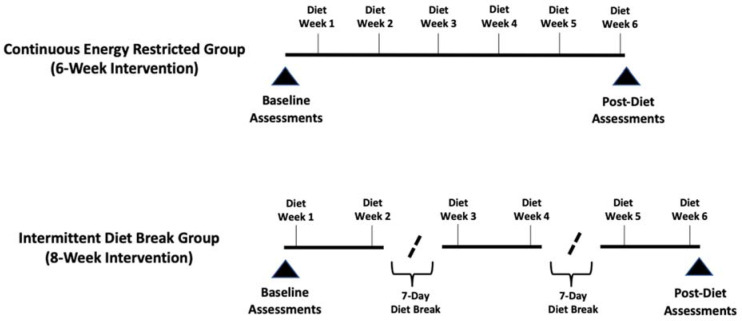
Timelines of energy restriction and energy balance for Intermittent and Continuous Energy Restriction groups.

This study utilized a parallel-groups, repeated-measures design wherein enrolled participants were assessed at baseline before allocation to a particular study group and matched according to body fat percentage. Matched participants were then randomized to either a six-week continuous energy-restricted diet (CON) or an intermittent energy-restricted diet (INT) that included a one-week period of energy balance after the second and fourth weeks of energy restriction. Both diets included a total of six weeks spent in a prescribed 25% energy deficit from predetermined weight maintenance levels; however, the INT group spent two additional weeks in energy balance throughout the intervention (Figure 1). Both groups participated in a supervised and volume-matched resistance training program. Primary outcomes were laboratory measures of body composition (fat-free mass [FFM], fat mass [FM], and body fat percentage) and resting metabolic rate (RMR). Secondary outcomes were measures of eating behavior and perceived hunger levels as well as home-based body composition data collected through commercially available bathroom scales utilizing bioelectrical impedance analysis.

### Participants

**Figure 2:**
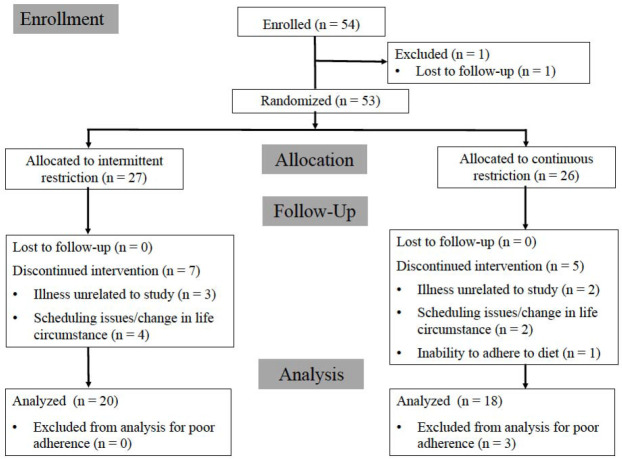
CONSORT flow diagram.

Participants were required to have at least six months of self-attested continuous resistance training experience leading up to the time of enrollment and to be free from metabolic disease. Of the 54 resistance-trained females initially enrolled, 38 participants (CON: n = 18; mean ± SD age 21.3 ± 3.8 years; body height 165.1 ± 7.1 cm; body mass 61.2 ± 7.4 kg; body fat content 24.7 ± 4.1%; resistance training experience 3.0 ± 2.5 years; INT: n = 20; mean ± SD age 23.3 ± 4.4 years; body height 162.4 ± 6.5 cm; body mass 64.2 ± 10.2 kg; body fat content 25.3 ± 4.7%; resistance training experience 2.7 ± 2.2 years) completed all aspects of the intervention and were included in the final per-protocol data analysis. A loss of at least 0.45 kg fat mass was determined before analysis as the primary screening criterion to further assess compliance issues, leading to the exclusion of three completers from the per-protocol analysis. In a small number of subjects, errors in the calculation of prescribed energy and macronutrient intake targets from baseline values were noted after analysis of the data. However, all participants retained in the per-protocol analysis reported an average energy deficit of at least 20% and/or lost at least 0.45 kg fat mass through the intervention. The additional 15 participants for whom baseline data were available were included in intent-to-treat (ITT) analyses. A CONSORT participant flow diagram, including reasons for attrition, is presented in Figure 2. Informed consent was obtained from all participants and the study was approved by the University of South Florida Institutional Review Board (Pro00039978) and was in compliance with the Declaration of Helsinki as revised in 1983.

### Measures

Throughout the study, participants uploaded daily nutritional data as tracked via a mobile app (MyFitnessPal, San Francisco, CA) to an online spreadsheet. Prior to baseline testing, participants were instructed to track their typical diet and body weight for 12–14 days to estimate their maintenance energy requirement. All participants were assigned a diet coach to assist the regular reporting of daily intake and home scale data and provide knowledge and support to participants thoughout the intervention. A pre-diet baseline testing session was scheduled during the participants' second week of tracking their maintenance calories. Before each laboratory visit, participants were instructed to fast for at least eight hours (an overnight fast) and refrain from physical activity for the previous 24 hours. After the diet period ended, a post-intervention assessment was scheduled that was identical to the pre-diet baseline testing assessment.

Upon entering the laboratory, participants urinated and then had their body height measured on a physician beam scale (Health-O-Meter; model 402KL; Pelstar, Inc., McCook, IL). Then, participants filled out two questionnaires. The first was a 51-item Three-Factor Eating Questionnaire (TFEQ; commonly referred to as the Eating Inventory, or EI) which assesses an individual’s level of dietary restraint, disinhibition, and hunger (Stunkard and Messick, 1985). In the small number of cases in which participants provided either no answer or ambigous answers that would affect the item's scoring (e.g., neither answered simply “true” nor “false”) on the questionnaire, the components affected by those missing or ambiguous answers were coded as missing data for that time point (representing 4.4% of all data points in *per protocol* and 5.3% of ITT). Second, participants filled out a five-item questionnaire which measured the following items on a seven-point Likert scale: hunger over the past seven days; fullness over the past seven days; desire to eat over the past seven days; ease of sticking to the diet for the past seven days; and motivation to diet for the week ahead. Landmarks on the Likert scale ranged from 1 (e.g., “not at all hungry”, “not at all motivated”) to 4 (e.g., “normal hunger”, “normal motivation”) to 7 (e.g., “extremely hungry”, “extremely motivated”). Participants also completed the same five-item questionnaire at the end of every third resistance training session so that changes in these measures could be tracked week-to-week.

Next, body mass and total body water were measured with multi-frequency bioelectrical impedance analysis (InBody® 570 Body Composition Analyzer, Biospace, Inc., Seoul, Korea). After the total body water measurement, resting metabolic rate (RMR) was assessed using a Parvo Medics' TrueOne® 2400 (ParvoMedics, Sandy, UT) integrated metabolic measurement system. The metabolic measurement system was calibrated prior to every assessment. RMR was assessed for twenty minutes as participants lay supine under a hood. The first five minutes were discarded and the remaining time of the test was averaged for the calculation of RMR (Horner et al., 2001). The calculated test–retest reliability for the metabolic measurement system had an intraclass correlation of 0.998, SEM of 25.6 kcals, and minimal detectable change of 71 kcals.

After RMR assessments were completed, body composition was assessed using the BodyMetrix™ BX-2000 A-mode ultrasound (IntelaMetrix, Livermore, CA) with a standard 2.5 MHz probe according to procedures as previously described, wherein fat thickness was measured at seven different sites and percent body fat, fat mass, and FFM were estimated based on the manufacturer's software (Colquhoun et al., 2017). All body composition assessments were completed by the same trained technician. The calculated body fat percentage test–retest reliability had an intraclass correlation of 0.993, SEM of 0.61%, and minimal difference of 1.69%.

### Design and Procedures

All participants were placed on a diet that prescribed a 25% reduction from their baseline energy intake and were instructed to consume 1.8 grams of protein per kilogram of baseline body mass. Remaining prescribed calories were distributed as 60% from carbohydrate and 40% from fat. To assist participants in both groups with the protein intake requirements, all participants were provided with whey protein isolate after each resistance exercise session (25 grams of ISO-100 from Dymatize Nutrition). For the two one-week periods in energy balance, participants in INT were instructed to consume their predetermined energy needs as assessed during baseline tracking and to continue to consume 1.8g/kg protein. All participants were provided with a home bathroom scale (RENPHO, Anaheim, CA) to record their daily body weight and body composition variables (estimated percent body fat and body water) throughout the intervention. The commercially available scales utilize foot-to-foot bioelectrical impedance analysis to provide estimates of body composition through a mobile application. Participants were instructed to weigh themselves each morning after using the bathroom and before ingesting food or liquid.

The resistance training program comprised three weekly supervised sessions throughout the duration of the study (six weeks for CON and eight weeks for INT) with an alternating upper body/lower body split. The INT group completed three sets per exercise per workout, while the CON group completed four sets per exercise per workout to account for the additional two weeks of intervention for the INT group such that total training volume would be matched. The program also followed a daily undulating periodization format, so that each workout cycled through three different repetition ranges of varying intensity: 4–12, 8–15, and 10–20. All working sets were completed within two repetitions in reserve. All but three of the 38 participants completed all assigned sessions, with these three participants missing one session each. A summary of the average weekly set volume for each major muscle group is provided in Table 1. Participants were instructed to engage in 30 min of low- to moderate-intensity aerobic activity twice per week. Participants in the INT group were told to refrain from aerobic exercise during their two week-long diet breaks.

**Table 1:** Average weekly set volume.

Muscle Group	INT (avg. # sets/week)	CON (avg. # sets/week)	% Total Volume*
Chest	4.5	6	6
Back	4.5	6	6
Shoulders	9	12	12
Triceps	13.5	18	18
Biceps	9	12	12
Quadriceps	9	12	12
Hamstrings	9	12	12
Glutes	18	24	24

Note: while average weekly set volume differed, total program training volume was equal for both diet groups due to the different lengths of the training program; * = percentage contribution of each muscle group to total workout volume.

### Statistical Analysis

All statistical tests were performed using a significance level of ⍺ = 0.05, and residuals for mixed models were extracted and visually screened for notable departures from normality. In the presence of significant main effects or interaction effects with more than two levels, pairwise post hoc comparisons were completed using the Tukey-Kramer method. Estimates from linear models are reported as estimated least square mean (LSMean) ± standard error unless otherwise noted, and estimates from robust ANOVAs are reported as trimmed mean ± trimmed standard error unless otherwise noted.

#### Per Protocol

Body composition, metabolic rate, and Three-Factor Eating Questionnaire scores from baseline and post-intervention were analyzed using a series of linear mixed models with random intercepts (SAS Version 9.4; SAS Institute Inc., Cary, NC). The participant was identified as a random effect, with fixed effects including the group, time (baseline versus post-testing), and the group × time interaction effect. The use of parametric tests for Likert scale items is controversial, but traditional non-parametric tests have drawbacks as well; as a result, five-item questionnaire items were analyzed using robust analysis of variance (ANOVA) procedures from the WRS2 package in R software (Version 3.5.1; R Foundation for Statistical Computing, Vienna, Austria). Factors included the group, time, and the group × time interaction effect, and robust ANOVA models were constructed using trimmed means with a 20% trimming level.

Secondary outcomes included weekly home-based scale data and were aligned based on the number of weeks in a deficit, such that both groups had six time points represented. Body composition data were averaged within each week and analyzed using linear mixed models, with fixed factors including the group, week, and the group × week interaction; correlated measures across weeks were modeled using a first-order autoregressive covariance structure. Five-item questionnaire outcomes were analyzed using robust ANOVA as described above, with factors including the group, the week, and the group × week interaction effect. Missing data for five-item questionnaire outcomes (two values during one week in one participant, and five values during one week in another) were imputed using the last observation carried forward method.

Tertiary outcomes included daily changes in body weight during diet breaks, measured at home. These data were analyzed using linear mixed models with random intercepts. The participant was identified as a random effect, with fixed effects including the diet break number (1 or 2, corresponding to the diet breaks occurring in week 3 and week 5 of the intervention), the day of diet break (1 through 7), and the break number × day interaction. Correlations among the days within a given diet break were modeled using a first-order autoregressive covariance structure. Body weight and body composition values for one participant on one day were missing and therefore excluded from this analysis; for body water, one day of data was entered in error and additional three days were missing across four participants. These observations were removed from the daily analyses.

An independent-samples *t*-test was used to identify any between-group differences in upper-body, lower-body, and total training tonnage (sets x repetitions x load) completed through the intervention. Independent-samples *t-*tests and Bonferroni-adjusted repeated-measures ANOVAs were used to identify differences in energy and macronutrient intake between groups and between different stages of the diet intervention (baseline, energy restriction, and diet break periods), respectively.

#### Intent-to-Treat

Intent-to-treat analyses were carried out using data from all participants who were allocated to a treatment and completed baseline testing. Intent-to-treat analyses were completed using linear mixed models as described above for pre- to post-intervention changes in body composition variables, metabolic rate, and Three-Factor Eating Questionnaire items (measured in the laboratory), and for weekly changes in body composition variables (using home-based scale measurements).

## Results

Dietary intake data are summarized in Table 2. There were no significant differences between groups for energy or macronutrient intake at baseline, both when expressed in absolute terms and relative to baseline body weight (all *p* ≥ 0.25). During energy restriction, a significant difference in fat and carbohydrate intake relative to bodyweight (g/kg/day) was observed between groups (*p* = 0.04 for both), though relative intake of protein was similar (*p* = 0.39). From baseline to energy restriction, both groups reported significant decreases in energy, fat, and carbohydrate intake, both when expressed as absolute measures and relative to body weight (all *p* ≤ 0.001), while measures of protein intake increased (*p* ≤ 0.004 for both). The level of reported energy restriction achieved while dieting was ~26% in INT and ~22% in CON. During the two week-long diet break periods, participants in INT significantly increased their absolute and relative intake of energy, fat, and carbohydrates (all *p* < 0.001), while protein intake remained consistent (*p* ≥ 0.88 for both). There were no significant differences between groups for upper-body, lower-body, and total training volume completed through the intervention (all *p* ≥ 0.11).

**Table 2:** Energy and macronutrient intake.

	INT		CON		
	Baseline	Diet	Diet Breaks	Baseline	Diet
Kcals	1,689 ± 368	1,245 ± 304*#	1,629 ± 400*	1,772 ± 414	1,379 ± 268*
CHO (grams)	193 ± 44	118 ± 40*#	178 ± 56*	205 ± 58	142 ± 38*
PRO (grams)	85 ± 29	110 ± 18*	113 ± 21*	83 ± 25	103 ± 15*
Fat (grams)	65 ± 13	37 ± 13*#	52 ± 16*	69 ± 20	44 ± 13*
Kcals/kg body mass	27 ± 6.4	20 ± 4.7*#	26 ± 6.4	29 ± 7.0	23 ± 4.7*
CHO (g/kg/day)	3.1 ± 0.7	1.9 ± 0.6*#	2.8 ± 0.9	3.4 ± 0.9	2.4 ± 0.7*
PRO (g/kg/day)	1.3 ± 0.5	1.7 ± 0.1*	1.8 ± 0.2*	1.4 ± 0.4	1.7 ± 0.2*
Fat (g/kg/day)	1.0 ± 0.2	0.6 ± 0.2*#	0.8 ± 0.3	1.1 ± 0.4	0.7 ± 0.2*
CHO/PRO/Fat (%)	46-20-34	38-35-27	43-28-29	46-19-35	41-30-29

Repeated Measures ANOVA, Bonferroni Adjusted. ^*^Significantly different than baseline (p < 0.05).

#Significantly different than Diet Breaks (p < 0.05). Relative values (kcals and g/kg) based on average body mass values from the home scale during the two-week baseline tracking period.

## Per Protocol

### Laboratory Measures

Body composition and RMR data are summarized in Table 3. Across all participants, there was a mean decrease in body weight from baseline to post-intervention, from 62.7 ± 1.5 kg to 61.5 ± 1.5 kg (*p* < 0.001). Body fat content decreased from 25.0 ± 0.8% to 23.5 ± 0.8% (*p* < 0.0001), and fat mass decreased from 15.9 ± 0.7 kg to 14.7 ± 0.7 kg (*p* < 0.0001). Fat-free mass did not change over time (from 46.8 ± 0.9 kg to 46.8 ± 0.9 kg; *p* = 0.90). There were no significant group-by-time interactions with regard to body weight, percent body fat, fat mass, and fat-free mass (all *p* ≥ 0.08). Across all participants, the RMR did not change over time (from 1422 ± 32 kcals to 1434 ± 32 kcals; *p* = 0.48) and the group-by-time effect for this variable was not significant (*p* = 0.99).

**Table 3:** Changes in body composition and resting metabolic rate (mean ± SD).

	INT				CON			
	Pre	Post	Change (CI)	ES	Pre	Post	Change (CI)	ES
BW (kg)	64.2 ± 10.2	62.4 ± 10.4	−1.7 (−2.6 – −0.8)	−0.93	61.2 ± 7.4	60.5 ± 7.8	−0.7 (−1.6 – 0.2)	−0.39
BF (%)	25.3 ± 4.7	23.9 ± 5.0	−1.4 (−2.1 – −0.7)	−0.86	24.7 ± 4.1	23.2 ± 4.8	−1.5 (−2.2 – −0.8)	−1.02
FM (kg)	16.6 ± 5.1	15.2 ± 5.1	−1.3 (−1.9 – −0.7)	−1.05	15.3 ± 3.9	14.2 ± 4.0	−1.1 (−1.6 – −0.6)	−1.13
FFM (kg)	47.6 ± 5.8	47.2 ± 6.2	−0.4 (−1.0 – 0.2)	−0.36	46.0 ± 4.6	46.3 ± 5.2	+0.4 (−0.4 – 1.2)	0.24
RMR (kcals / day)	1,431 ± 217	1,443 ± 187	+12 (−37 – 61)	0.12	1,412 ± 169	1,425 ± 198	+12 (−42 – 66)	0.11

BW = body weight; BF = body fat; FM = fat mass; FFM = fat-free mass; RMR = resting metabolic rate; CI = 95% confidence interval using S.E. of mean change from paired-samples t-test; ES = effect size for paired samples (Cohen's Dz), calculated as $\frac{t}{\sqrt{N}}$

Overall, five-item questionnaire values increased over time for hunger (3.96 ± 0.11 to 4.29 ± 0.13; *p* = 0.04) and desire to eat (4.12 ± 0.12 to 4.92 ± 0.23; *p* < 0.01). Scores did not change over time for measures of satiety, ease of sticking to the diet, or motivation to diet for the week ahead (all *p* ≥ 0.06). There were no significant group-by-time interactions for any of these five variables from either baseline to post-intervention (all *p* ≥ 0.11) or week-to-week (all *p* ≥ 0.17).

For measures collected through the Three-Factor Eating Questionnaire (TFEQ), the level of dietary restraint did not change from baseline to post-intervention (from 12.12 ± 0.63 to 12.55 ± 0.63; *p* = 0.28), nor did the level of hunger (from 4.67 ± 0.53 to 4.97 ± 0.53; *p* = 0.40). While there were no differences between groups for change over time in these variables (*p* = 0.51 and *p* = 0.17, respectively), there was a significant group-by-time interaction for disinhibition (*p* < 0.01). While no pairwise comparisons were significantly different, a divergent pattern was observed, with mean values for disinhibition generally increasing from baseline to post-intervention in CON (from 4.91 ± 0.73 to 6.17 ± 0.71; *p* = 0.11), but decreasing in INT (from 6.80 ± 0.68 to 6.05 ± 0.68; *p* = 0.42).

## Home-Based Measures

Overall, weekly average fat mass and percent body fat decreased over time with a significant effect for week as measured with the home scale (*p* < 0.001 and *p* = 0.01, respectively) with no significant group-by-time interaction (*p* ≥ 0.24 for both). There was a group-by-time interaction for change in body mass (*p* = 0.02) and FFM (*p* < 0.01). Body mass and FFM decreased significantly over time in INT, with average weekly values from diet weeks 2-6 all lower than week 1 (*p* < 0.001 and *p* < 0.01, respectively). However, there were no statistically significant changes within CON for body mass (all *p* ≥ 0.84) or FFM (all *p* ≥ 0.87) from week 1 to any subsequent week.

**Figure 3:**
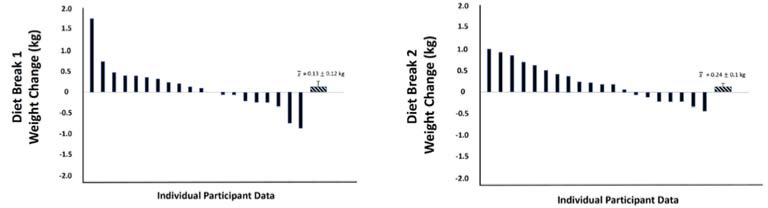
Changes in weekly average body weight during diet break weeks.

During the two diet break weeks in INT, there was a significant effect for time on body mass, with values increasing by a mean of 0.53 kg from day 1 to day 7 (*p* < 0.01). Body fat content also increased by a mean of 0.2 points from day 1 to 7 (*p* = 0.02). Percent body water generally decreased over the latter half of the week, with a change of -0.16 points from day 4 to 7 (*p* = 0.01). Changes for these three variables did not significantly differ between the first and second diet breaks (all *p* ≥ 0.09), and there were no interaction effects between the diet break number and the day of diet break (all *p* ≥ 0.47). The mean changes in weekly average body mass values from the week prior to the week of the diet break were a gain of 0.13 kg for the first diet break and 0.24 kg for the second diet break. However, there was notable variability between individuals, with some losing up to 0.82 kg and some gaining up to 1.68 kg between weeks. Figure 3 summarizes individual-level changes in weekly average body mass values from the week prior to the week of diet breaks 1 and 2.

## Intent-to-Treat

### Laboratory Measures

When all 53 participants who were initially randomized were analyzed together, the interaction effect was statistically significant for body mass, with INT losing 1.2 kg more than CON (*p* = 0.04). However, for all body composition variables (FFM, FM, and percent body fat) and RMR, there were no significant differences between groups over time, in line with the findings of the per-protocol analysis (all *p* ≥ 0.09). Similarly, the group-by-time effect for disinhibition remained significant (*p* = 0.01), whereas the interaction effects for restraint (*p* = 0.39) and hunger (*p* = 0.13) remained non-significant.

### Home-Based Measures

The group-by-week interaction effect for body mass observed in the per-protocol analysis maintained its significance in ITT, with INT losing more body weight week-to-week (*p* = 0.01). Additionally, the interaction effect for FFM remained, with INT losing more FFM than CON (*p* < 0.01). Values for FM generally decreased from week 1 to 6 of energy restriction by an average of 0.30 kg (*p* = 0.03), with no significant group-by-time interaction (*p* = 0.19).

### Discussion

#### Body Composition and RMR

The primary finding of this investigation was that six weeks of intermittent (INT) dieting at a prescribed 25% reduction in energy intake presented no improvements in body composition or RMR when compared with continuous (CON) energy restriction. In terms of total weight loss, lean body mass retention, and metabolic measures, the current body of research suggests that intermittent energy restriction is at least comparable to continuous energy restriction (Davis et al., 2016; Harris et al., 2018; Peos et al., 2021) with some studies even suggesting that it may provide unique benefits (Byrne et al., 2018; Davoodi et al., 2014; Varady, 2011). However, the vast majority of literature on intermittent energy restriction has focused on more extreme forms of energy intake undulation such as alternate-day fasting (Seimon et al., 2015), and many do not involve intermittent periods in which a return to true energy balance is both prescribed and achieved – that is, in most studies of intermittent energy restriction to date, either spontaneous or formal energy restriction is continued to some extent throughout the “feast” periods (Sainsbury et al., 2018).

The specific nature of the intermittent energy restriction utilized in the current investigation was designed to reflect a “diet break” approach, wherein periods of energy restriction are interspersed with periods of neutral or positive energy balance lasting four or more consecutive days (Escalante et al., 2020). Existing research on this approach is sparse, with four of the five existing trials to our knowledge conducted in subjects with overweight or obesity (Arguin et al., 2012; Byrne et al., 2018; Keogh et al., 2014; Wing and Jeffery, 2003). These investigations employed cumulative duration of energy restriction ranging from 14 to 26 weeks. Data from Byrne and colleagues (2018) suggest that intermittent energy restriction in males with obesity may attenuate metabolic adaptations to energy restriction and improve fat loss efficiency over 16 cumulative weeks of a prescribed 33% reduction in energy intake. The remaining three trials in untrained populations showed no notable benefits of this approach, although it should be noted that one study (Keogh et al., 2014) did not match participants for total time instructed to diet, meaning that the intermittent restriction group experienced similar weight loss over one year while only instructed to diet at ~1,315 kcals per day (approximately a 23% reduction from reported baseline intake) for half of the time (26 weeks) of the continuous group (52 weeks). However, Arguin and colleagues (2012) reported a greater decrease in lean body mass in sedentary post-menopausal women with obesity who dieted intermittently compared to those dieting continuously over 15 cumulative weeks at approximately 22% energy restriction from baseline.

The sole study to our knowledge conducted in a resistance-trained population demonstrated no appreciable difference between continuous and intermittent energy restriction on changes in body composition or resting energy expenditure over 12 cumulative weeks of approximately 34% energy restriction from baseline (Peos et al., 2021). However, while our study also examined the effect of alternating periods of a similar degree of energy restriction (~24%) with week-long periods of energy balance in a resistance-trained population, it is unique in that it was conducted in conjunction with a standardized and supervised resistance training program. Our findings echo the majority of previous studies in both trained and untrained populations and suggest that diet breaks have no notably beneficial effects on body composition and metabolic rate.

In consideration that our participants were not obese, perhaps a more appropriate comparison would be to dieting physique athletes (who also engage in resistance exercise and consume relatively high amounts of dietary protein when in an energy deficit, which are characteristics shared with participants in the present study). While no controlled intervention trials have been conducted on competitive physique athletes, there are reports of physique athletes adopting intermittent energy restriction in preparation for competition (Chappell et al., 2018; Mitchell et al., 2018; Trexler et al., 2014). Physique athletes may incorporate intermittent energy restriction strategies with the intent to offset some of the negative consequences of dieting reported in lean individuals, including losses of lean body mass (Garthe et al., 2011), suppression of muscle protein synthesis (Pasiakos et al., 2010), increases in rates of muscle protein breakdown (Carbone et al., 2014), and anabolic resistance (Murphy and Koehler, 2020). It is important to note that female physique athletes who undergo energy restriction while engaging in resistance and aerobic exercise typically go to greater efforts to lose fat mass than resistance-trained females in our investigation. Female physique ahletes typically adhere to hypoenergetic diets for 18-30 or more weeks and reach relative energy deficits greater than 40% (Petrizzo et al., 2017; Rohrig et al., 2017; Tinsley et al., 2019).

In lean, resistance-trained individuals, prior research reported that metabolic adaptations and losses of fat-free mass in response to energy restriction manifest under conditions of severe energy deficits undertaken for extended periods of time or when dietary protein intake levels are kept low (Chappell et al., 2021; Garthe et al., 2011; Mettler et al., 2010; Rohrig et al., 2017; Tinsley et al., 2019). Neither of these conditions were met in the current investigation as the energy deficit was approximately 24% for a six-week period, and protein intakes averaged 1.7 g/kg/d, which is within the recommended range for active individuals looking to build or preserve muscle mass (Jager et al., 2017). If the energy deficit, duration of the diet, and reductions in protein intake are not severe enough to induce metabolic adaptations and a loss of FFM, a diet break (or any other strategy employed) will have limited to no utility for improving metabolic adaptations or adverse physiologic outcomes that the prescribed diet failed to induce. A recent examination of intermittent versus continuous energy restriction in resistance-trained subjects found that resting energy expenditure decreased significantly in both groups after 12 weeks of approximately 34% energy restriction with no differences between groups at the end of the intervention (Peos et al., 2021). It is possible that more notable changes in metabolic rate may have been observed in our cohort if participants had continued to diet for several additional weeks, but this is not likely, given that small but statistically non-significant increases were seen in both groups over six weeks of dieting.

It should be noted that participants in INT generally lost FFM as estimated via ultrasound, while participants in CON generally gained FFM, although changes over six weeks of energy restriction were not significantly different within or between groups. Furthermore, data collected through home-based scales indicated a loss of FFM with intermittent compared to continuous energy restriction. Although these data are likely less accurate than the laboratory measures used, our findings overall suggest that diet breaks do not attenuate loss of FFM in the population studied, nor do they have a notably beneficial impact on FM loss or RMR retention. It is uncertain whether the FFM changes detected via ultrasound would reach statistical significance if the intervention lasted for longer than six weeks, as found by a similar investigation in untrained post-menopausal women dieting for 15 cumulative weeks and assessed by dual-energy x-ray absorptiometry (Arguin et al., 2012). It is also possible that the bioelectrical impedance analysis technology of the commercially available scales used by participants at home was not sensitive to small changes in body composition, and thus attributed the greater loss of body weight in INT to loss of FFM rather than fat mass.

Another potential reason for these findings is the design of the resistance training program. In order to equate both total time spent dieting and total training volume completed over the course of the intervention, participants in INT completed 18 fewer sets per week than participants in CON (54 sets per week for 8 weeks in INT versus 72 sets per week for 6 weeks in CON for a total of 432 sets prescribed). Current scientific consensus on the impact of training volume on muscle growth suggests that higher weekly set volumes elicit a greater hypertrophic response, with one landmark meta-analysis suggesting that weekly set volumes of 10 or greater can contribute to an additional 3.2% increase in growth compared to 5–9 weekly sets, although this effect was reduced to 1.4% when one particularly influential study was removed in a sensitivity analysis (Schoenfeld et al., 2017). In the present study, participants in INT completed between 4.5 and 18 average sets per muscle group per week, while participants in CON completed between 6 and 24; thus, it is not inconceivable that an overall greater weekly set volume in CON allowed for improved retention of FFM over the course of the diet, or at least diminished the potential FFM-sparing effects of the diet breaks. Recent research in a similar resistance-trained population also found no beneficial effect of intermittent energy restriction on FFM retention over 12 weeks of dieting, though training volume was not monitored or reported (Peos et al., 2021). Future research on intermittent energy restriction in resistance-trained populations should aim to determine the effects of this approach on body composition and RMR measures when weekly set volume, rather than total sets completed, are equated, and over a duration longer than six weeks.

#### Hunger and Eating Behavior

While seven of the eight variables we assessed related to hunger and eating behavior showed no effect of intermittent versus continuous energy restriction, there was a significant effect for disinhibition, with values generally increasing over time in CON and decreasing over the same period in INT. Previous research in a similar population as ours suggests that decreased disinhibition during a weight loss intervention predicts successful weight loss over the course of a year (Cheng et al., 2014). Additionally, research in post-menopausal women has demonstrated that “dieters” (those who reported currently trying to lose weight) tended to have higher disinhibition scores as well as a higher body mass index (BMI) than “non-dieters”, even if they also scored high for dietary restraint (Rideout and Barr, 2009). In other words, the “diet mindset”, which is associated with a higher BMI and increased disinhibition, is conceptually distinct from a “restraint mindset” applied independently of dieting, which is associated with a lower BMI. It is possible that by nature of the ability to “practice” restrained, but not excessively restrictive, eating throughout the two week-long diet break periods, participants in the INT group saw fewer deleterious effects of dieting on disinhibition than those dieting continuously, which may bode well for the maintenance of energy balance and weight loss results over the long term. However, we found no significant effect of the diet breaks on the participants’ self-reported ease of sticking to the diet or motivation to continue the diet for the duration of our study.

Previous research of Wing and Jeffery (2003) showed that participants assigned to take two-week breaks after every three weeks of dieting showed sharp upticks in body weight during the first and second, but not the third diet break, despite the fact that they reported a higher intake of highly palatable, energy-dense “target foods” during all three breaks compared to those dieting continuously at analogous timepoints. Thus, it is possible that subjects were able to gain practice eating these foods, which were deemed off-limits during the active dieting periods, in a way that promoted self-regulation of energy balance over time and attenuated the impact of prolonged energy restriction on disinhibition. Together, these findings mirror previous research demonstrating the beneficial effects of “flexible” versus “rigid” forms of restraint on weight loss maintenance (Sairanen et al., 2014). While the present study found significant increases in subjective hunger and desire to eat across participants after six weeks of dieting, recent research in a similar population reported greater hunger and desire to eat in the continuous group at the end of the intervention, indicating a potentially beneficial effect of intermittent restriction; however, the effect was not consistent, as differences in ratings of fullness or prospective consumption did not occur (Peos et al., 2021). Future research is needed to further investigate the specific relationship between intermittent energy restriction, measures of appetite and eating behaviors, and long-term weight loss maintenance.

#### Strengths and Limitations

While the difference in weekly set volumes between groups is one potential limitation of our findings, it must be balanced with the effort to equate for the total number of sets completed over the course of the intervention in both groups. In addition, our group of participants was recreationally trained, self-attesting to at least six months of continuous resistance training experience before entering the study; however, most were not considered athletes, followed a wide variety of training styles before enrollment, and no strength-based standards were required for entry. It is also possible that participants engaged in additional exercise outside the three supervised resistance and two self-directed aerobic training sessions per week, but we did not elicit data regarding compliance in this regard.

Strengths of our study include the fact that nearly all training sessions were directly supervised in our laboratory, that each participant was assigned a personal “diet coach” to field questions and provide guidance throughout the intervention, and that participants received a home scale to weigh themselves daily. It is possible that all of these aspects of the study improved adherence and retention by providing a sense of accountability, motivation, and personal connection with research staff and fellow participants. We were able to retain 77% of initially randomized subjects, of whom 93% were deemed adequately compliant with the nutrition protocol. In addition, our performance of ITT analysis of key variables allowed us to assess and reasonably discard any concerns of attrition bias. Finally, our study included measures of self-reported hunger and eating behavior variables, which further elucidate the potential effect of intermittent energy restriction on the psychological in addition to the physiological impacts of energy restriction on weight loss maintenance.

### Conclusions

The findings of our study suggest that in resistance-trained females seeking to optimize their physiques, the use of diet breaks within the context of six weeks of a prescribed 25% reduction in energy intake does not improve the efficiency of fat loss and has no beneficial effect on FFM and RMR. Intermittent dieting strategies may be employed for those who desire a short-term break from an energy-restricted diet without fear of fat regain. Additionally, though the use of diet breaks did not appear to affect changes in hunger or dietary restraint over six weeks of energy restriction, it may reduce the impact of prolonged energy restriction on measures of disinhibition, potentially helping to promote increased long-term dietary adherence. However, any such potential benefit should be weighed against the cost of spending more total time on the diet—time that could otherwise be spent continuing to lose fat or transitioning back to energy balance or a surplus sooner.
